# The occurrence of comorbidities with affective and anxiety disorders among older people with intellectual disability compared with the general population: a register study

**DOI:** 10.1186/s12888-019-2151-2

**Published:** 2019-06-03

**Authors:** Nadia El Mrayyan, Jonas Eberhard, Gerd Ahlström

**Affiliations:** 1 Department of Health Sciences, Faculty of Medicine, Lund University, PO Box 157, SE-22100 Lund, Sweden; 2 Division of Psychiatry, Clinical Psychosis Research Unit, Region Skane and Affiliated to Department of Clinical Sciences, Lund University, SE-25187 Helsingborg, Sweden

**Keywords:** Depression, Developmental disability, Intellectual disability, Mood disorders, Learning disabilities, Prevalence, Comorbidity

## Abstract

**Background:**

Little is known regarding the burden of comorbidities among older people with intellectual disability (ID) who have affective and anxiety disorders. Therefore, we aimed to investigate the occurrence and risk of psychiatric and somatic comorbidities with affective and/or anxiety disorders in older people with ID compared to the general population.

**Methods:**

This population study was based on three Swedish national registers over 11 years (2002–2012). The ID group was identified in the LSS register, which comprises of data on measures in accordance with the Act Concerning Support and Service for Persons with Certain Functional Impairments (*n* = 7936), and a same-sized reference cohort from the Total Population Register was matched by sex and year of birth. The study groups consisted of those with affective (*n* = 918) and anxiety (*n* = 825) disorder diagnoses. The information about diagnoses were collected from the National Patient Register based on ICD-10 codes.

**Results:**

The rate of psychiatric comorbidities with affective and anxiety disorders was approximately 11 times higher for people with ID compared to the general reference group. The two most common psychiatric comorbidities occurred with affective and anxiety disorders were Unspecified non-organic psychosis and Other mental disorders due to brain damage and dysfunction and to physical disease (8% for each with affective disorders and 7 and 6% with anxiety disorders, respectively). In contrast, somatic comorbidity comparisons showed that the general reference group was 20% less likely than the ID cohort to have comorbid somatic diagnoses. The most commonly occurring somatic comorbidities were Injury, poisoning and certain other consequences of external causes (49 and 47% with affective and anxiety disorders, respectively) and Signs and symptoms and abnormal clinical and laboratory findings not elsewhere classified (44 and 50% with affective and anxiety disorders, respectively).

**Conclusion:**

Older people with ID and with affective and anxiety diagnoses are more likely to be diagnosed with psychiatric comorbidities that are unspecified, which reflects the difficulty of diagnosis, and there is a need for further research to understand this vulnerable group. The low occurrence rate of somatic diagnoses may be a result of those conditions being overshadowed by the high degree of psychiatric comorbidities.

## Background

Depression and anxiety are significant public health issues that affect older people and present a great burden for individuals, families and society [1]. These conditions may cause an increased burden for people in the highly vulnerable group with intellectual disability (ID) due to their communication difficulties [2]. It is acknowledged that the percentage of older people in the world is growing rapidly, both in general and in regards to individuals with ID [3]. With increased age comes ageing-related diseases, which add burden to ID-related illnesses that emerge during early childhood as well as those that develop in adulthood [4]. Thus, older people with ID suffer from advanced and complex somatic and psychiatric comorbidities, and accurate diagnosis and management can be difficult due to the person’s decreased ability to understand and express his or her illness, leading to inappropriate service and care [4, 5]. The World Health Organization (WHO) reports that depression and anxiety are two of the most common mental disorders affecting older people, with 7 and 3.8% of the world’s older population being affected, respectively [1]. Furthermore, depression and anxiety are considered to be common disorders in individuals with ID, and they frequently occur together [6, 7].

However, the results from studies of the general population cannot be directly generalized to older people with ID because there are major differences between the groups [8]. The communication deficits that limit the ability of people with IDs to describe and report their symptoms to health care providers result in unsatisfactory clinical consultations and poor treatment choices [9–11]. These problems may increase with the severity of the ID and limit the appropriate diagnosis of affective and anxiety disorders [7, 12, 13]. A Dutch study compared the prevalence of depression and anxiety in older people with ID to that in the general population, and it reported that depression and anxiety disorders increase with age and are more common among people with ID than they are in the general population [7]. Additionally, psychiatric conditions such as affective and anxiety disorders may be associated with a higher risk of other psychiatric and somatic diseases [6]. Our research group investigated the occurrence of psychiatric diagnoses in a specialist care setting among older people with ID in relation to the general population. We found that people with ID had more than double the risk of affective disorders (OR = 1.74) and anxiety disorders (OR = 1.36) [14]. In this study, we considered comorbidities to understand the disease burden of older people with ID who also have affective and/or anxiety disorders.

Understanding the differences in the diagnoses and comorbidities of older people with ID compared to the general population is important for developing appropriate policy strategies and reducing differences in health care interventions [8]. While there has been an increase in the literature on the health issues of people with ID, strong epidemiological studies on a population level with large sample sizes and appropriate diagnostic criteria are still needed to identify the occurrence rates of most common disorders as they relate to affective and anxiety diagnoses for older people with ID [4]. Moreover, to the best of our knowledge, there have been no recent studies investigating the occurrence rates of affective and anxiety disorders with other psychiatric and somatic comorbidities in older people with ID compared to the general population. Therefore, the aim of this study was to investigate the co-occurrence and risk of psychiatric and somatic comorbidities with affective and/or anxiety disorders in older people with intellectual disability compared to people of the same age and sex in the general population without ID.

## Methods

This study is a retrospective population study from Sweden based on register data from three national registers over 11 years.

### Swedish national registers used in the study

1) The LSS register is based on a supportive measure that comes from the Act Concerning Support and Service for Persons with Certain Functional Impairments (LSS) [15]. The LSS law gives people with significant and permanent functional impairments or disabilities the right to receive special support and services with the purpose of providing them with living conditions equal to those experienced by individuals without these disabilities. The LSS register contains three groups; individuals having intellectual disability, autism or resembling autism (Person group 1); individuals having intellectual disability as a result of permanent brain damage in adulthood (Person group 2); finally, individuals having other physical or mental impairment that is clearly not due to normal aging (Person group 3). This study included Person group 1, which applies to individuals with intellectual disability, autism or autism spectrum disorders [15].

2) The Swedish National Patient Register (NPR register) was established in 1987, and it requires the mandatory registration of inpatient and outpatient specialist care patients. It contains information about medical data, listing one main diagnosis and up to 21 secondary diagnoses [16]. In this study, we identified individuals who had at least one diagnosis of affective and/or anxiety disorders with other comorbidities. The diagnostic information is coded according to the International Classification of Diseases (ICD-10) codes. The National Board of Health and Welfare is the authority responsible for both the LSS and NPR registers.

3) The Swedish Total Population Register (TPR register) was created in 1968, and it contains information about Sweden’s general population. In the TPR register, data are maintained by Statistics Sweden, which is the official source for population statistics [17].

### Study groups

#### ID study group

The study group comprised individuals with ID, autism, and autism spectrum disorders (Person group 1), as identified in the LSS register. The ID group was selected from an identified population aged 55 years and older that was alive and living in Sweden as of December 31, 2012 (*n* = 7936). The definition of older age in this study (55 years and older) was based on previous research showing that people with ID age earlier than the general population [18]. Of those with ID, we included individuals who were also diagnosed with affective (F3) and/or anxiety (F4) disorders, as coded according to the ICD-10 and collected from the NPR register over the course of eleven years (2002–2012).

#### General reference group (gRef)

**Fig. 1:**
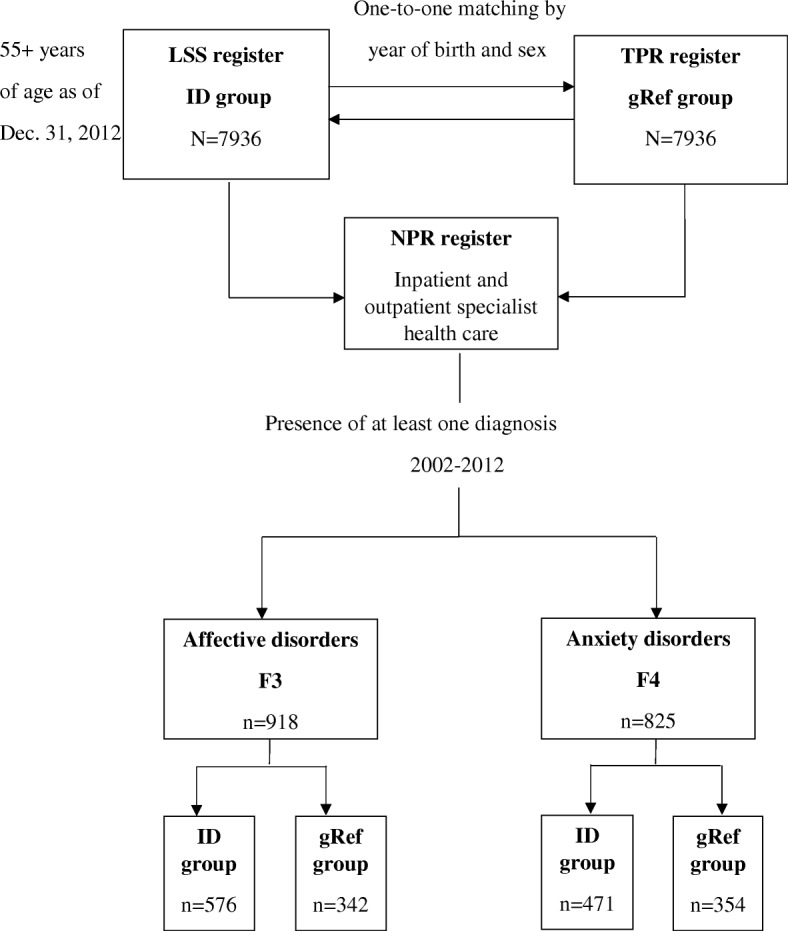
Flow chart on the sampling procedure of the study group

The second study group was selected from the general population via one-to-one matching to each case in the ID population (*n* = 7936) by sex and year of birth during the same time period (2002 to 2012). The matching procedure was performed by Statistic Sweden. This study group (gRef) was similar to the ID study group in that its members had at least one diagnosis of affective and anxiety disorders. Figure 1 shows the procedure used for sampling from the three registers and the number of cases diagnosed with each affective (F3) and anxiety (F4) disorder in the older people with ID group and in the gRef group. The total study group consisted of 1743 people in both cohorts.

Affective disorders are disorders caused by a change in the affect or mood of the person (ICD-10, 2016). Most affective disorders are recurrent, and the onset of episodes can be related to stressful situations (ICD-10, 2016). Anxiety disorders are stress-related disturbances that cause significant maladaptation in social, occupational or personal function (ICD-10, 2016). The subdivisions of affective and anxiety diagnoses (using two-digitICD-10 codes) are shown in Table 1. The percentage of individuals with at least one affective disorder was higher among the older people with ID compared to the gRef (*n* = 576, 7.3% and *n* = 352, 4.3%, respectively). Depressive episode disorders (F32), Bipolar affective disorders (F31) and Recurrent depressive disorders (F33) were the most common affective disorders in the ID group. Furthermore, the percentage of individuals with at least one anxiety disorder was higher among people with ID compared to the gRef (*n* = 417, 5.9% and *n* = 354, 4.5%, respectively). The most common anxiety disorders were Other anxiety disorders (F41) and Reaction to severe stress and adjustment disorders (F43).

**Table 1:** The affective and anxiety disorders in the study groups of older people with intellectual disability (ID) (7936) and in the general population from the same cohort (7936), from 2002 to 2012^a^

	ID *n* = 7936		gRef *n* = 7936	
	n	%	n	%
Affective Disorders (F3)				
(F30) Manic episode	32	0.4%	<5	0.0%
(F31) Bipolar affective disorder	170	2.1%	42	0.5%
(F32) Depressive episode	358	4.5%	254	3.2%
(F33) Recurrent depressive disorder	116	1.5%	116	1.5%
(F34) Persistent mood [affective] disorders	21	0.3%	23	0.3%
(F38) Other mood [affective] disorders	< 5	0.0%	5	0.1%
(F39) Unspecified mood [affective] disorder	35	0.4%	17	0.2%
At least one F3	576	7.3%	342	4.3%
Anxiety Disorders (F4)				
(F40) Phobic anxiety disorders	36	0.5%	16	0.2%
(F41) Other anxiety disorders	289	3.7%	196	2.5%
(F42) Obsessive-compulsive disorder	74	0.9%	10	0.1%
(F43) Reaction to severe stress, adjustment disorders	81	1.0%	143	1.8%
(F44) Dissociative [conversion] disorders	27	03%	<5	0.0%
(F45) Somatoform disorders	31	0.4%	48	0.6%
(F48) Other neurotic disorders	11	0.1%	5	0.1%
At least one F4	471	5.9%	354	4.5%

^a^Reference: “Psychiatric diagnoses in older people with intellectual disability in comparison with the general population: a register study” by Axmon, A., Björne, P., Nylander, L., & Ahlström, G. (2017), Epidemiology And Psychiatric Sciences, 1–13. 10.1017/S2045796017000051

#### Outcome measure

We identified all other psychiatric and somatic comorbidities that were found in the NPR register between 2002 and 2012 by using the ICD-10 diagnoses. The psychiatric comorbidities consisted of all diagnoses in the mental and behavioural chapter, excluding affective and anxiety disorders (F3, F4), behavioural and emotional disorders with onset usually occurring in childhood and adolescence (F9), disorders of psychological development (F8) and intellectual disability (F7) because they were included in the selected study groups.

The included psychiatric comorbidities were based on the ICD-10 block subdivisions with 2 digits (i.e., F20 Schizophrenia, F60 Specific personality disorders) because affective and anxiety disorders are included in the same chapter of mental and behavioural diagnoses. The somatic comorbidities were considered separately from the psychiatric diagnoses in this study; therefore, they are presented as ICD-10 chapter levels (i.e., II Neoplasms, VI Diseases of the nervous system). All somatic comorbidities were included except those in chapter XXI, Factors influencing health status and contact with health services, as that chapter contains information about health care services and is not a diagnosis of comorbidities.

### Statistical analysis

In addition to the descriptive statistics, logistic regression was used to estimate the odds ratio and 95% confidence intervals to assess whether age and sex were significantly associated with at least one affective and anxiety diagnosis in the ID group and the gRef group. To compare the occurrence rates and the risk of having psychiatric and somatic comorbidities with affective and anxiety disorders in the ID and gRef groups, logistic regression was used to estimate the odds ratio with 95% confidence intervals. Regarding comorbidities, values less than 5 were not reported. Statistical analysis was performed using IBM SPSS Statistics version 24.0.

## Results

The age range of the patients included in the study was 55 to 96 years as of December 31, 2012. As shown in Table 2, the younger age groups were more likely than the oldest age groups to have at least one affective or anxiety diagnosis. Furthermore, logistic regression showed that the odds ratio was higher for the ID group to have at least one affective or anxiety diagnosis, but the relationship was not statistically significant in any age group compared to individuals who were less than 64 years old. More females than males were diagnosed with affective and anxiety disorders, except in the ID group, where the males were diagnosed with at least one anxiety diagnosis (*n* = 247, 52% and *n* = 224, 48%, respectively) (Table 2). Furthermore, among individuals with at least one anxiety disorder, the odds ratio for the ID group was significant only for females (OR = 0.6, 95% CI 0.5–0.8), Table 2. For the ID group with affective and/or anxiety diagnoses, the result shows that the occurrence of psychiatric comorbidities is approximately 11 times higher for older people with ID compared to the general study group (Table 3). In contrast, the comparison of somatic comorbidities showed 80% more somatic diagnoses in the general reference group (gRef) than in the ID group.

**Table 2:** Age and sex of older people with intellectual disability (ID) and a reference sample from the general population (gRef), stratified by the presence of at least one diagnosis of affective or anxiety disorder

Age in 2012 and sex	At least one F3			At least one F4		
	*n* = 918			*n* = 825		
	ID *n* = 576	gRef *n* = 342	OR (95% CI)	ID *n* = 471	gRef *n* = 354	OR (95% CI)
Age, n (%)						
<64	376 (65%)	219 (64%)	Reference^*^	342 (73%)	248 (70%)	Reference^*^
65–74	172 (30%)	87 (25%)	2.5 (0.7–9.2)	112 (24%)	81 (23%)	1.8 (0.4–8.2)
75–84	24 (4%)	30 (9%)	2.9 (0.8–10.7)	14 (3%)	21 (6%)	1.8 (0.4–8.4)
>84	4 (1%)	6 (2%)	1.2 (0.3–4.7)	3 (1%)	4 (1%)	0.8 (0.17–4.5)
Sex, n (%)						
Male	279 (48%)	165 (48%)	Reference ^*^	247 (52%)	150 (42%)	Reference^*^
Female	297 (52%)	177 (52%)	0.9 (0.7–1.2)	224 (48%)	204 (58%)	**0.6 (0.5–0.8)**

^*^Reference

Note. Statistically significant ORs are marked in bold

**Table 3:** Occurrence of at least one psychiatric comorbidity and somatic comorbidity with at least one affective (F3) and anxiety (F4) diagnosis among older people with intellectual disability (ID) and the general population (gRef)

Comorbidity	At least one F3 diagnosis (*n* = 918)			At least one F4 diagnosis (*n* = 825)		
	ID (*n* = 576) n(%)	gRef (*n* = 342) n(%)	ID v gRefOR (95% CI)	ID (*n* = 471) n(%)	gRef (*n* = 354) n(%)	ID v gRefOR (95% CI)
At least one psychiatric comorbidity	520 (90%)	159 (47%)	**10.69 (7.55–15.14)**	414 (88%)	139 (39%)	**11.23 (7.92–15.94)**
At least one somatic comorbidity	479 (83%)	294 (86%)	0.81 (0.55–1.17)	391 (83%)	302 (85%)	0.84 (0.58–1.23)

Note. Statistically significant ORs are marked in bold

### Psychiatric comorbidities

Table 4 summarizes the occurrence rates of psychiatric comorbidities in patients with at least one affective and/or anxiety diagnosis in the ID group and the gRef based on two categories of Mental and behavioural disorders in Chapter V. The most common comorbidities in the ID group with affective diagnoses were Other mental disorders due to brain damage and dysfunction and to physical disease (F06), Unspecified nonorganic psychosis (F29) and Specific personality disorders (F60) (8%, *n* = 44; 8%, n = 44; and 6%, *n* = 35, respectively). As shown in Table 4, the higher risk was only statistically significantly higher for F06 (OR = 3.45, 95% CI 1.61–7.43) and F23 (OR = 3.06, CI 1.16–8.07) comorbidities with at least one affective diagnosis in the ID group.

**Table 4:** Occurrence of psychiatric comorbidities with at least one affective (F3) and anxiety (F4) diagnosis among older people with intellectual disability (ID) and the general population (gRef) based on two-digit mental and behavioural diagnoses from the ICD-10 coding system

Psychiatric comorbidity	At least one F3 diagnosis (*n* = 918)			At least one F4 diagnosis (*n* = 825)		
	ID (*n* = 576) n(%)	gRef (*n* = 342) n(%)	ID v gRef OR (95% CI)	ID (*n* = 471) n(%)	gRef (*n* = 354) n(%)	ID v gRef OR (95% CI)
F00_Dementia in Alzheimer disease	5 (1%)	<5	NC*	<5	<5	NC*
F01_Vascular dementia	11 (2%)	<5	NC*	<5	<5	NC*
F03_Unspecified dementia	29 (5%)	8 (2%)	2.21 (1.00–4.90)	13 (3%)	<5	NC*
F06_Other mental disorders due to brain damage and dysfunction and to physical disease	44 (8%)	8 (2%)	**3.45 (1.61–7.43)**	27 (6%)	7 (2%)	**3.01 (1.30–7.00)**
F07_Personality and behavioural disorders due to brain disease, damage and dysfunction	10 (2%)	<5	NC*	9 (2%)	<5	NC*
F09 _Unspecified organic or symptomatic mental disorder	6 (1%)	<5	NC*	6 (1%)	<5	NC*
F10_Mental and behavioural disorders due to use of alcohol	34 (6%)	53 (16%)	**0.34 (0.22–0.54)**	32 (7%)	46(13%)	**0.49 (0.31–0.79**)
F11_Mental and behavioural disorders due to use of opioids	6 (1%)	11 (3%)	**0.32 (0.12–0.86)**	<5	10 (3%)	NC*
F13_Mental and behavioural disorders due to use of sedatives and hypnotics	<5	22 (6%)	NC*	< 5	21 (6%)	NC*
F17_Mental and behavioural disorders due to use of tobacco	13 (2%)	13 (4%)	0.58 (0.27–1.28)	10 (2%)	13 (4%)	0.57 (0.25–1.31)
F19_Mental and behavioural disorders due to multiple drug use and use of other psychoactive substance	10 (2%)	17 (5%)	**0.34 (0.15–0.77)**	8 (2%)	15 (4%)	**0.39 (0.16–0.93)**
F20_Schizophrenia	25 (4%)	<5	NC*	29 (6%)	<5	NC*
F22_Persistent delusional disorders	22 (4%)	6 (2%)	2.22 (0.89–5.54)	20 (4%)	<5	NC*
F23_Acute and transient psychotic disorders	25 (4%)	5 (2%)	**3.06 (1.16–8.07)**	19 (4%)	5 (1%)	**2.93 (1.09–7.94)**
F25_Schizoaffective disorders	17 (3%)	<5	NC*	14 (3%)	<5	NC*
F28_Other nonorganic psychotic disorders	<5	<5	NC*	5 (1%)	<5	NC*
F29_Unspecified nonorganic psychosis	44 (8%)	<5	NC*	34 (7%)	<5	NC*
F51_Nonorganic sleep disorders	<5	9 (3%)	NC*	<5	6 (2%)	NC*
F60_Specific personality disorders	35 (6%)	14 (4%)	1.52 (0.80–2.86)	35 (7%)	14 (4%)	**1.95 (1.03–3.68)**
F63_Habit and impulse disorders	5 (1%)	<5	NC*	6 (1%)	<5	NC*

*NC (not calculated) indicates that one cell contains a value that is less than 5 or that there were zero observations

Note. Statistically significant ORs are marked in bold

Among those with at least one anxiety diagnosis in the ID group, the most common comorbidities were Specific personality disorders (F60), 7%; Unspecified nonorganic psychosis (F29), 7%; and Other mental disorders due to brain damage and dysfunction and to physical disease (F06), 6% (n = 35, *n* = 34, and *n* = 27, respectively). These comorbidities were similar to those seen with the affective diagnosis, as shown above. However, Mental and behavioural disorders due to the use of alcohol (F10) were the most common psychiatric comorbidities in the general reference group (gRef), with at least one affective diagnosis in 16% (*n* = 53) and with at least one anxiety diagnosis in 13% (*n* = 46), Table 4. A significantly higher risk of having psychiatric comorbidities with at least one anxiety diagnosis in the ID group was observed in patients with F06 (OR = 3.01, CI 1.30–7.00), F23 (OR = 2.93, CI 1.09–7.94) and F60 (OR = 1.95, CI 1.03–3.68) comorbidities (Table 4).

### Somatic comorbidities

As summarized in Table 4, somatic comorbidities were categorized based on the chapters of the ICD-10 coding system. The most common comorbidities with affective diagnoses in the ID group were Injury, poisoning and certain other consequences of external causes (49%, Chapter XIX); Symptoms, signs and abnormal clinical and laboratory findings, not elsewhere classified (44%, Chapter XVIII); and Diseases of the digestive system (33%, Chapter XI), (*n* = 280, *n* = 251, *n* = 188, respectively). Moreover, there was a significantly higher risk of being diagnosed with a disease of the nervous system (Chapter VI, OR = 1.66, CI = 1.20–2.28), a disease of the genitourinary system (Chapter XIV, OR = 1.41, 95% CI = 1.01–1.96) or Injury, poisoning and certain other consequences of external causes (Chapter XIX, OR = 1.37, CI = 1.04–1.79) in the ID group with at least one affective diagnosis (Table 5).

**Table 5:** Occurrence of somatic comorbidities with at least one affective (F3) and anxiety (F4) diagnosis among older people with intellectual disability (ID) and the general population (gRef) based on the chapters of the ICD-10 coding system

Chapter	Somatic comorbidity	At least one F3 diagnosis (*n* = 918)			At least one F4 diagnosis (*n* = 825)		
		ID (*n* = 576) n(%)	gRef (*n* = 342) n(%)	ID v gRef OR (95% CI)	ID (*n* = 471) n(%)	gRef (*n* = 354) n(%)	ID v gRef OR (95% CI)
I	Certain infectious and parasitic diseases	68 (12%)	34 (10%)	1.21 (0.79–1.87)	52 (11%)	25 (7%)	1.63 (0.99–2.67)
II	Neoplasms	71 (12%)	53 (16%)	0.77 (0.52–1.13)	39 (8%)	48 (14%)	**0.58 (0.37–0.90)**
III	Diseases of the blood and blood-forming organs and certain disorders involving the immune mechanism	41 (7%)	31 (9%)	0.77 (0.47–1.25)	23 (5%)	24 (7%)	0.71 (0.39–1.27)
IV	Endocrine, nutritional and metabolic diseases	168 (29%)	112 (33%)	0.85 (0.63–1.12)	120 (26%)	91 (26%)	0.99 (0.72–1.36)
VI	Diseases of the nervous system	170 (30%)	69 (20%)	**1.66 (1.20–2.28)**	138 (29%)	60 (17%)	**2.03 (1.44–2.86)**
VII	Diseases of the eye and adnexa	117 (20%)	62 (18%)	1.15 (0.82–1.62)	89 (19%)	52 (15%)	1.35 (0.93–1.97)
VIII	Diseases of the ear and mastoid process	24 (4%)	16 (5%)	0.89 (0.46–1.70)	22 (5%)	22 (6%)	0.74 (0.40–1.36)
IX	Diseases of the circulatory system	159 (28%)	151 (44%)	**0.49 (0.36–0.64)**	134 (29%)	130 (37%)	**0.69 (0.51–0.92)**
X	Diseases of the respiratory system	149 (26%)	104 (30%)	0.80 (0.60–1.07)	130 (28%)	109 (31%)	0.86 (0.63–1.16)
XI	Diseases of the digestive system	188 (33%)	133 (39%)	0.76 (0.58–1.01)	172 (37%)	136 (38%)	0.92 (0.69–1.23)
XII	Diseases of the skin and subcutaneous tissue	37 (6%)	17 (5%)	1.31 (0.73–2.37)	26 (6%)	16 (5%)	1.23 (0.65–2.33)
XIII	Diseases of the musculoskeletal system and connective tissue	126 (22%)	134 (39%)	**0.44 (0.32–0.58)**	124 (26%)	141 (40%)	**0.54 (0.40–0.72)**
XIV	Diseases of the genitourinary system	143 (25%)	65 (19%)	**1.41 (1.01–1.96)**	125 (27%)	58 (16%)	**1.84 (1.30–2.61)**
XVII	Congenital malformations, deformations and chromosomal abnormalities	39 (7%)	< 5	NC*	31 (7%)	< 5	NC*
XVIII	Symptoms, signs and abnormal clinical and laboratory findings, not elsewhere classified	251 (44%)	170 (50%)	0.79 (0.60–1.02)	236 (50%)	182 (51%)	0.95 (0.72–1.25
XIX	Injury, poisoning and certain other consequences of external causes	280 (49%)	140 (41%)	**1.37 (1.04–1.79)**	220 (47%)	133 (38%)	**1.46 (1.10–1.93)**

*NC (not calculated) indicates that one cell contains a value that is less than 5 or that there were zero observations

Note. Statistically significant ORs are marked in bold

Furthermore, the most common comorbidities with at least one anxiety diagnosis were Symptoms, signs and abnormal clinical and laboratory findings, not elsewhere classified (50%, Chapter XVIII, *n* = 236); Injury, poisoning and certain other consequences of external causes (47%, Chapter XIX, *n* = 220); and Diseases of the digestive system (37%, Chapter XI, *n* = 172). In the general study group, Symptoms, signs and abnormal clinical and laboratory findings, not elsewhere classified (Chapter XVIII), was the most common somatic comorbidity in patients with at least one affective diagnosis (50%, *n* = 170) and at least one anxiety diagnosis (51%, *n* = 182) (Table 5).

In patients with at least one anxiety diagnosis, there was a significantly higher risk of having a diagnosis of Disease of the nervous system (Chapter VI, OR = 2.03, CI = 1.44–2.86), Disease of the genitourinary system (Chapter XIV, OR = 1.84, CI = 1.30–2.61) or an Injury, poisoning and certain other consequences of external causes diagnosis (Chapter XIX, OR = 1.46, CI = 1.10–1.93), Table 5.

## Discussion

The findings of this study show that the odds of being diagnosed with one psychiatric comorbidity was eleven times higher among older people with ID and affective and/or anxiety disorders compared to the general population. Older people with ID and affective and/or anxiety disorders have a higher rate of unspecified psychiatric and somatic comorbidities than the general population, which indicates that people with ID are a more vulnerable group that presents an evident need for collaboration between health and social services [19, 20]. Moreover, we found that the most common psychiatric and somatic comorbidities were similar for patients with affective and anxiety disorders. This finding can be explained by the fact that mood disorders such as depression and anxiety are associated with one another [21]. Regarding somatic comorbidities, we found that the ID group was approximately 20% less likely to have a comorbid somatic diagnosis with affective and anxiety disorders than the general population.

### Psychiatric comorbidities

Among the findings of this study, it is interesting to note that the most common psychiatric comorbidities are unspecified or categorized as other disorders. These findings align fairly well with those of Baxter et al. [22] that people with ID are at risk of having an unidentified diagnosis; this further supports the clinical experience that it is difficult for health care professionals to recognize health problems in patients with severe and profound types of ID and psychiatric comorbidities. In addition, the health care provider often attributes the symptoms of the psychiatric disorders to ID symptoms, when in fact the problems are actually related to a comorbid psychiatric diagnosis. The main factor contributing to health care providers’ low level of knowledge regarding people with ID and psychiatric comorbidities was a lack of sufficient training and experience in the assessment and treatment of people with ID and with psychiatric disorders [23, 24]. As a consequence, the presence of affective and anxiety disorders with other comorbidities might be hidden and thus underestimated by health care providers [25].

In addition, patients with more severe ID have a limited ability to describe their symptoms [26–28]. Furthermore, the calming effect of the psychotropic medications commonly used for individuals with ID may hide the symptoms of psychiatric disorders and make it more difficult for health care providers to diagnose comorbidities [29–31]. This may suggest an increased vulnerability among older people with ID.

We found a significantly lower risk of substance abuse comorbidities, such as alcohol, opioid, smoking and psychoactive drug abuse in older people with ID, which is in line with what McCarron et al. [5] reported in their study of multimorbidity in older people with ID. In contrast, other studies have found that a higher rate of alcohol misuse in individuals with ID [32, 33]. These contradictory results can be explained by the different conditions, settings and characteristics of the studies’ sample populations, e.g., younger age groups are at an increased risk of substance abuse; patients with mild-to-moderate ID are at higher risk of substance abuse than those with more severe ID; and the inclusion of primary care data may affect results as people are more often treated for alcohol misuse in that setting.

### Somatic comorbidities

Regarding somatic comorbidities, older people with ID and affective and/or anxiety disorders had a lower percentage of somatic comorbidities than the general population. However, the results showed a higher risk of diseases of the nervous system or genitourinary system and injury/poisoning in older people with ID with affective and anxiety disorders.

Furthermore, our results regarding the higher occurrence rates of diseases of the neurological and digestive systems within the ID group support previous reports [5]. For example, in a previous study, comorbid epilepsy, constipation, and dyspepsia [33] occurred more frequently in individuals with ID. However, in our study, Diseases of the digestive system occurred more frequently in the general population than in older people with ID. A possible explanation might be that health care providers miss the diagnosis and symptoms of the digestive system, and therefore, less-severe somatic problems are not detected.

Moreover, our findings share some similarities with previous studies that show lower rates of cardiovascular, cancer, and pulmonary disease [5, 33, 34]. The rates of cardiovascular disease increase significantly with age in the general population, but in our study, cardiovascular disease was less prevalent in older people with ID and affective and/or anxiety disorders. One possible explanation consistent with previous research is under diagnosis or unidentified diagnoses in the ID population [35]. Another explanation for the lower rates of cardiovascular disease in our study could be related to the lower rates of substance use (i.e., smoking and alcohol) in our population. Lower cardiovascular and stroke rates have been previously reported to be associated with light drinking and less smoking among older people [36].

Furthermore, this study found that the risk of injuries and poisoning is higher in people with affective and/or anxiety disorders. This indicates that older people with ID and affective and/or anxiety disorders are more vulnerable and prone to injuries. Therefore, our recommendation is to develop strategies and policies to promote health and prevent injuries in older people with ID. Cox et al. (2010) found several risk factors for injuries and falls in people with ID. These risk factors include hypertension, visual impairment, polypharmacy and the use of psychotropic medications [37]. Older people are also more likely to be prescribed multiple medications, such as antipsychotics and benzodiazepine, which are associated with a wide range of side effects [36, 38]. Additionally, studies report that the use or misuse of prescribed benzodiazepine and prescription sedatives in older people has been associated with an increased risk of falls [39, 40].

This study shows that patients with ID and anxiety and depression are less frequently diagnosed with signs and symptoms of somatic diseases and are less likely to have abnormal clinical findings compared to the general population. However, when compared with the prevalence of the other somatic comorbidities in the ID group, signs, symptoms and abnormal clinical findings were the second-most prevalent comorbidities. Some signs and symptoms, such as headache, musculoskeletal pain and pain related to the circulatory and respiratory systems, were less likely to be diagnosed in older people with ID [41]. This may be because those symptoms require good communication skills to relay them to health care providers, and individuals with ID may not be able to describe their problem sufficiently [41]. Furthermore, pain related to the urinary system is more likely to be diagnosed in older people with ID because it is easier to diagnose using laboratory tests and cultures [41]. These findings can explain our study results regarding the more frequent occurrence of diseases of the genitourinary system among older people with ID compared to the general population.

Previous research regarding comorbidities in older people with ID has reported a lower occurrence of cardiovascular diseases [5] and a lower risk of being diagnosed with musculoskeletal and cardiovascular pain [41]. Older people with ID need good communication skills to describe their symptoms to health care providers when accessing health care. This may explain why diseases of the musculoskeletal and circulatory systems are less likely to be reported in older people with ID with affective and/or anxiety disorders compared to the general population.

Jakovljevic (2009) showed that people with comorbid mental or somatic disorders experience problems receiving care both because some psychiatrists fail to recognize somatic diseases and because somatic specialists do not recognize mental disorders, and therefore they do not provide adequate treatment [42]. The comorbid occurrence of mental disorders with other psychiatric and somatic disorders is also presented in a recently growing body of literature about multimorbidity in the ageing population [43–45]. For example, a pattern of multimorbidity was identified by the presence of relationships between mental and neurological diseases and gastrointestinal and mental and neurological diseases in older people with ID [5] This could contribute to a better understanding of the complexity of comorbidities in people with ID and affective and anxiety disorders [43].

### Methodological considerations

This paper provides information about all psychiatric and somatic diagnoses that co-occur with affective and anxiety disorders in older people with ID in inpatient and outpatient specialist care. Our study was based on national register data coded by ICD-10 profiles in the ID population and general population, while previous studies focused on a single or small number of comorbidities, used self-reported data and did not make any comparison with the general population [8]. Another strength of this study is the high validity of the NPR registry, which is based on inpatient and outpatient care data and has required mandatory registration for physicians for more than 30 years [46]. Furthermore, studies with data from the NPR registry regarding diagnoses with affective and anxiety disorders such as bipolar, obsessive compulsive disorders and tic disorders have more than 90% positive predictive value [47–50]. Additionally, using both the LSS register, which was developed specifically for people with intellectual disability, and the Swedish total population register is an advantage of this study because both data sources have mandatory registration and high population coverage.

The registers used in the study were designed as administrative registers; thus, they minimize the ability to observe the individuals in the study and thereby identify other possible risk factors that could affect the diagnosis by health care providers. Another limitation is the lack of information from the primary health care provider, which limits information about comorbidities as the NPR register does not include any data about primary health care. Data files about primary health care in Sweden are collected on the county level and were often started in the last decade by the 21 county councils. However, the Swedish government plans to make a decision that will include data on primary health care in the NPR. The current absence of national data needs to be taken into consideration given that affective and anxiety disorders are usually treated in the primary health care setting. One recent study based on data from the Primary Care Register in nine counties included 72% of the Swedish population and reported that 80% of depression and anxiety disorders were diagnosed only by the primary health care provider [51]. This result confirms that depression and anxiety are more often diagnosed in the primary health care setting. However, that study focused on the general population, and it is unknown whether the pattern is the same for people with ID.

In the present study, we investigated the occurrence of affective and anxiety disorders with other comorbidities without taking into consideration the level of ID in our study population. The risk of multimorbidity has been previously reported to increase with the severity of ID [52]. ID severity can complicate the assessment of clinical manifestations and the diagnosis of other psychiatric disorders and can increase the length of stay in a medical facility [8, 53]. Also, with severe level of ID, the utility of different diagnostic criteria used in people with ID becomes very limited because is based on verbal expression. The diagnosis of psychiatric disorders in people with severe to profound levels of ID is more complicated because the symptoms of the disease are usually masked by behavioural disturbances, which lead to problems in the identification and treatment of additional diagnoses [54]. Finally, we chose to analyse different levels of diagnostic specificity for somatic and psychiatric comorbidities due to our focus on psychiatric diagnoses in this study. This should be taken into consideration when interpreting the results regarding somatic diagnoses.

## Conclusion

This study indicated that older people with ID and affective and/or anxiety disorders are approximately eleven times more likely to be diagnosed with at least one another psychiatric comorbidity compared with the general population without ID. This study highlighted the high occurrence rates of other and unspecified psychiatric comorbidities and the low occurrence rates of somatic diagnoses in this study group of older people with ID. The findings suggest that more attention should be paid to comorbidities in older patients with affective and/or anxiety disorders and ID. More in-depth knowledge is needed regarding whether comorbidities generate an increased burden for older people with ID. Future research can therefore focus on health care utilization for ageing people with ID and other comorbidities.

## Data Availability

This study contains sensitive information about a vulnerable group of people with intellectual disability. Although the data files are anonymized, they still contain details that enable the possible identification of single individuals. If access to the database is requested for other researchers, the PI (Gerd Ahlström) would have to ask the Regional Ethical Review Board in Lund before could provide the data. This is due to considerable restrictions regarding access to the data that were placed on the study prior to approval. However, the database was compiled by three national registers, which other researchers can recreate by contacting the Swedish National Board of Health and Welfare and Statistics Sweden.

## Abbreviations

gRef: General Reference Group
ICD-10: International Classification of Diseases-10th Revision
ID: Intellectual Disability
LSS: The Act Concerning Support and Service for Persons with Certain Functional Impairments
NPR: National Patient Register
TPR: Total Population Register
